# A Sabadilla Case

**Published:** 1887-12

**Authors:** J. Feild Deck

**Affiliations:** Sydney, New South Wales


### A SABADILLA CASE.

J. FEILD DECK, M. D., SYDNEY, NEW SOUTH WALES.

Mrs. R., a lady about twenty-eight years of age, consulted me on account of the following symptoms, from which she had been suffering more or less for about three months. She could only ascribe them to some extra strain, mental and physical, to which she had been exposed. There is a tendency to neurotic disease in her family; one brother has lately died in an asylum, and another has shown symptoms of mental weakness. She has been living up the country, away from any medical advice of which she cared to avail herself, but she has been making use of simple hydropathic measures occasionally.

She complains of attacks of giddiness, which affect her in two different ways. At times attacks of short duration affect her very suddenly, in which everything seems to be whirling round her; she has to lay hold of something, otherwise she would fall down; if she is in the house, the whole house seems to be coming down upon her; if she is in the street, the houses around seem to be falling upon her, and unless she can lay hold of any support at once she falls down; she sometimes wakes at night with the same whirling sensation, and feels impelled to cling to her husband. These attacks come on without any warning, but only last a few minutes, but are followed by a weak, tired feeling.

At other times she suffers from attacks of much longer duration, sometimes lasting the greater part of the forenoon, and accompanied by nausea and some visual disturbance. Her brain seems to go round and round, and her eyes to move to and fro, as if they went round with the whirling sensation. If she shuts her eyes the whirling seems to go round in the opposite direction and she becomes sick. She likes to lie perfectly still, and to look very fixedly at one object; if she turns her eyes to look at anything else, or if she shuts her eyes, she becomes sick.

She feels worse in the mornings, very weak in the forenoon, and better in the afternoon; the sudden giddy attacks come on at any time, the attacks with nausea mostly in the mornings. She has a depressed, anxious look. Her appetite is very poor; likes milk, feels soon satisfied; has some nausea after meals, but no giddiness, flatulence, or distention.

She says her mouth is in a fearful state when she wakes, very dry and burning, the tongue dry and thickly coated; she wakes with a very bad taste, and drinks at that time, not because she is thirsty, but to cleanse her mouth. Her hands are affected in a strange way; the palms of the hands have become dry and horny, and inclined to scale; at night they are so burning that she has to keep them outside the bedclothes. The top of her head is hot; she feels best out-of-doors.

I gave her Pulsatilla[6] four times a day, and told her to stop the enemas she had been in the habit of using twice a day.

A week later her tongue was cleaner, her appetite better, but there was no change in the nervous symptoms. In one attack she had fallen down and hurt herself so much that she dreaded to go out. I could not find out that she was ever, even for a moment, unconscious during these attacks, nor had she ever passed water unconsciously at night; but I could not help telling her husband that I was apprehensive, considering the neurotic tendency in the family, that the attacks might eventuate in epilepsy.

In looking over her symptoms I was reminded of a key-note to Sabadilla amongst some collected by Carleton Smith in the first volume of THE HOMŒOPATHIC PHYSICIAN—headache better from looking fixedly at some object. My patient had laid so much stress upon the necessity she felt to look fixedly upon some object, that I felt this symptom might lead to a right selection of the remedy. With great interest I turned to the pathogenesis of Sabadilla, and there found such a full picture of her condition, including both subjective and objective symptoms, that I gave her Sabadilla[6] four times a day, and awaited her return in ten days' time with some degree of, I may say, excitement. Great was my pleasure when she returned to find that the distressing attacks of vertigo had nearly passed away. Under the same remedy, continued for another week, she returned to the country in three weeks' time quite recovered, and when I last heard, two months afterward, there had been no relapse in her condition.

As Hughes remarks, the list of symptoms of nearly every medicine contained in Jahr's *Codex* begins with vertigo, and I confess this *embarras de richesses* has often distressed me, and that in treating cases of vertigo I have studied Kafka's exhaustive article in the thirty-first volume of the *British Journal of Homœopathy* and Lilienthal's list of medicines in his *Homœopathic Therapeutics*, and that the only result has been a sense of bewilderment. Thankful was I, therefore, to Carleton Smith that this peculiar key-note (which I found in the pathogenesis of Sabadilla, and which was not therefore merely the result of clinical observation) had guided me to a medicine which I felt sure, if there was anything in Homœopathy, must act beneficially.

The rapid relief under the action of Sabadilla shows it to be an important medicine in disturbances of the sensorium, associated with gastric irritation, when there are also present dryness and burning of the mouth and dryness and burning of the palms of the hands. Pulsatilla is often indicated in gastric vertigo, but the aggravation of Pulsatilla is in the afternoon and evening, that of Sabadilla in the forenoon and night. Both have want of thirst, but there is desire for milk under Sabadilla, aversion to it under Pulsatilla. We find under Pulsatilla many kinds of vertigo, but not vertigo as if everything were turning, which is marked in Sabadilla. Under this remedy, too, we find, as a marked objective symptom, dryness of the hands during the whole time of the proving, worse in the forenoon, and some roughness of the skin; there is no parallel to this under Pulsatilla. And, lastly, we do not find any symptom under Pulsatilla analogous to that which proved a key-note to the use of Sabadilla. Constant headache or a sort of tension, less violent when staring at a thing or reflecting upon a thing. My patient suffered from vertigo improved by staring at a thing, but vertigo and the headache, which is described as a sort of tension, must be very nearly related as disturbances of the sensorium.

Since attending this case I have prescribed for a lady suffering from attacks of giddiness coming on generally in the evening. In one attack she fainted right off, then became sick and threw up food and bile; during the attacks she felt suddenly as if she would fall if she did not lay hold of something; she felt glad to get into the open air. The attacks were recent, associated with some gastric disturbance, as she felt sick after meals. Sabadilla$^{6}$ cured her in a few days.

*May* 17*th*, 1887.

